# Neurodegeneration-associated mitochondrial proteins, CHCHD2 and CHCHD10–what distinguishes the two?

**DOI:** 10.3389/fcell.2022.996061

**Published:** 2022-09-09

**Authors:** Aya Ikeda, Yuzuru Imai, Nobutaka Hattori

**Affiliations:** ^1^ Department of Neurology, Juntendo University School of Medicine, Tokyo, Japan; ^2^ Department of Research for Parkinson’s Disease, Juntendo University Graduate School of Medicine, Tokyo, Japan; ^3^ Research Institute for Diseases of Old Age, Graduate School of Medicine, Juntendo University, Tokyo, Japan; ^4^ Center for Genomic and Regenerative Medicine, Graduate School of Medicine, Juntendo University, Tokyo, Japan; ^5^ Neurodegenerative Disorders Collaborative Laboratory, RIKEN Center for Brain Science, Saitama, Japan

**Keywords:** genetics, mitochondria, amyothophic lateral sclerosis, parkinson's disease, dopaminergic (DA) neuron, motor neurons

## Abstract

Coiled-coil-helix-coiled-coil-helix domain containing 2 (CHCHD2) and Coiled-coil-helix-coiled-coil-helix domain containing 10 (CHCHD10) are mitochondrial proteins that are thought to be genes which duplicated during evolution and are the causative genes for Parkinson’s disease and amyotrophic lateral sclerosis/frontotemporal lobe dementia, respectively. CHCHD2 forms a heterodimer with CHCHD10 and a homodimer with itself, both of which work together within the mitochondria. Various pathogenic and disease-risk variants have been identified; however, how these mutations cause neurodegeneration in specific diseases remains a mystery. This review focuses on important new findings published since 2019 and discusses avenues to solve this mystery.

## 1 Introduction

Mitochondria are key organelles that maintain cellular metabolism and activity. Mitochondrial functions include adenosine triphosphate (ATP) production via oxidative phosphorylation (OXPHOS), biogenesis of phospholipids and amino acids, urea cycle reactions, the formation of heme and iron–sulfur clusters, apoptosis, and calcium homeostasis (Rahman and Rahman, 2018). Neurodegenerative diseases are characterized by selective degeneration of specific neurons, with mitochondrial damage and dysfunctions playing a role in some cases. Many studies have discovered a link between mitochondrial dysfunctions and neurodegenerative diseases such as Parkinson’s disease (PD), Huntington’s disease, amyotrophic lateral sclerosis (ALS), and Alzheimer’s disease (AD) (Johri and Beal, 2012).

*Coiled-coil-helix-coiled-coil-helix domain containing 10 (CHCHD10)* (MIM#615903) has been identified as a causative gene in a large French family with autosomal dominant inheritance for patients with heterogeneous symptoms, including motor neuron disease, cerebellar syndrome, and cognitive decline (Bannwarth et al., 2014). CHCHD10 is localized in the mitochondrial intermembrane space and is more abundant at cristae junctions (Bannwarth et al., 2014). Overexpression of the disease-associated CHCHD10 S59L causes mitochondrial fragmentation, loss, disassembly, and expansion of mitochondrial cristae (Bannwarth et al., 2014). In contrast, CHCHD10 protein levels were significantly lower in the spinal cord of patients with ALS and the frontal cortex of patients with frontotemporal lobe dementia (FTD) (McCann et al., 2020).

Following this discovery, *CHCHD2* (MIM#616244) mutations were identified as a pathogenic gene for patients with familial PD (Funayama et al., 2015). The autopsied brain of one of the CHCHD2 T61I patients revealed widespread Lewy body pathology, and phosphorylated *α*-synuclein accumulation was observed in dopaminergic neurons differentiated from induced pluripotent stem (iPS) cells obtained from another PD patient with CHCHD2 T61I (Ikeda et al., 2019). CHCHD2 variants in Caucasian patients with Lewy body disease were concentrated in the N-terminus, which contained a putative mitochondrial targeting sequence (MTS) (Ogaki et al., 2015). An exploratory study of PD biomarkers reported that CHCHD2 protein and mRNA were significantly reduced in the erythrocytes and substantia nigra of patients with PD and mice overexpressing *α*-synuclein A53T. However, the physiological significance of the reduction in CHCHD2 mRNA of erythrocytes is unclear as mature erythrocytes lose their mitochondria and nuclei (Liu et al., 2021).

This review aimed to discuss the pathomechanisms by which *CHCHD2* and *CHCHD10* mutations cause specific neurodegenerations, and recent key findings on the molecular functions of CHCHD2 and CHCHD10.

## 2 *CHCHD2* and *CHCHD10* are associated with neurodegenerative diseases

*CHCHD2* mutations were first identified in a Japanese PD family with dominant inheritance (Funayama et al., 2015). Three mutations (p.T61I, p. R145Q, and c.300 + 5G > A) have been identified as causal genes of PD. The patients with CHCHD2 T61I had unique parkinsonism symptoms such as a good response to levodopa in the absence of cognitive decline or dysautonomia over 10 years from the onset. Our pathological analysis revealed that a patient with CHCHD2 T61I had Lewy body pathology, matching Braak stage 6 (Ikeda et al., 2019). *Drosophila* models expressing human *α*-synuclein and human CHCHD2 T61I in a *Drosophila* ortholog of *CHCHD2* and *CHCHD10*, *CG5010* (also known as *C2C10H*)^*−/−*^ background reproduced parkinsonism-like phenotypes such as motor disabilities, shortened life span, and *α*-synuclein aggregation in the brain (Ikeda et al., 2019). Afterwards, numerous *CHCHD2* variants were reported in patients with dementia with Lewy bodies, PD, AD, and multiple system atrophy (Table 1).

**TABLE 1:** Disease-associated variants of *CHCHD2* and *CHCHD10*.

*Protein*	Mutation	Type of Mutation	Location	Ethnicity	Disease	References
CHCHD2	P2L (c. 5C > T)	Hetero	MTS domain	Japanese	Sporadic PD	Foo et al. (2015)
				Chinese	Early-onset PD and sporadic PD	Shi et al. (2016)
				Chinese	Sporadic PD	Funayama et al. (2015)
				Chinese	AD	Che et al. (2018)
				Caucasian	Sporadic DLB and PD	Ogaki et al. (2015)
	G4R (c.10G > A)	Hetero	MTS domain	Caucasian	DLB	Ogaki et al. (2015)
	S5R (c. 15C > G)	Hetero	MTS domain	Chinese	AD	Che et al. (2018)
	R8H (c. 23G > A)	Hetero	MTS domain	Japanese	Sporadic PD	Ikeda et al. (2017)
	P14S (c.40C > T)	Hetero	MTS domain	Caucasian	Sporadic PD	Ogaki et al. (2015)
	A16A (c.48C > T)	Hetero	MTS domain	Caucasian	Sporadic PD	Ogaki et al. (2015)
	R18Q (c.53G > A)	Hetero	MTS domain	Chinese	Sporadic PD	Yang et al. (2016)
	V31V (c.93C > T)	Hetero	MTS domain	Caucasian	Sporadic PD	Ogaki et al. (2015)
	A32T (c. 94G > A)	Hetero	MTS domain	Western	Early-onset PD	Jansen et al. (2015)
				European ancestry		
				Chinese	AD	Che et al. (2018)
	P34L (c. 101C > T)	Hetero	MTS domain	Western	Early-onset PD	Jansen et al. (2015)
				European ancestry		
				Caucasian	DLB and PD	Ogaki et al. (2015)
	A37V (c.110C > T)	Hetero	MTS domain	Caucasian	DLB	Ogaki et al. (2015)
	A49V (c.146C > T)	Hetero	MTS domain	Caucasian	Sporadic PD	Ogaki et al. (2015)
	T61I (c.182C > T)	Hetero	Conserved	Japanese	Autosomal dominant PD	Funayama et al. (2015)
			α-helix			
				Chinese	Autosomal dominant PD	Shi et al. (2016)
	V66M (c.196G > A)	Hetero	Conserved	Italian	MSA	Nicoletti et al. (2018)
			α-helix			
	A71P (c.211G > C)	*Homo*	Conserved	Caucasian	Early-onset PD	Lee et al. (2018)
			α-helix			
	A79S (c.235G > T)	Hetero	Conserved	Chinese	Sporadic PD	Yang et al. (2019)
			α-helix			
	I80V (c.238A > G)	Hetero	Conserved	Western	Early-onset PD	Jansen et al. (2015)
			α-helix	European ancestry		
	S85R (c.255T > A)	Hetero	Disordered region	Chinese	FTD	Che et al. (2018)
	A93V (c.278C > T)	Hetero	Disordered region	Caucasian	DLB	Ogaki et al. (2015)
	Q126X (c.376C > T)	Hetero	CHCH domain	German	Early-onset PD	(Eva Koschmidder, 2016)
	R145Q (c.434G > A)	Hetero	C-terminus	Japanese	Autosomal dominant PD	Funayama et al. (2015)
CHCHD10	P12S (c.34C > T)	Hetero	MTS domain	Spanish	ALS	Dols-Icardo et al. (2015)
	R15L (c.44C > A)	Hetero	MTS domain	German	Autosomal dominant ALS	(Muller et al., 2014)
				United States	Familial ALS	Johnson et al. (2014)
				German	Autosomal dominant motor neuron disease	Kurzwelly et al. (2015)
				Caucasian	Sporadic ALS	Zhang et al. (2015)
	R15S (c.43C > A)	Hetero	MTS domain	Hispanic	Autosomal dominant mitochondrial myopathy	Ajroud-Driss et al. (2015)
	H22Y (c.64C > T)	Hetero	Disordered region	Chinese	Sporadic FTD	Jiao et al. (2016)
	P23S (c.67C > T)	*Homo*	Disordered region	Chinese	Sporadic FTD	Jiao et al. (2016)
	P23L (c.68C > T)	Hetero	Disordered region	Chinese	Sporadic FTD	Shen et al. (2017)
	P24L (c.71G > A)	Hetero	Disordered region	Chinese	FTD	Xiang-Qian Che, (2017)
	S30L (c.89C > T)	Hetero	Disordered region	Chinese	Sporadic PD	Zhou et al. (2018)
	A32D (c.95C > A)	Hetero	Disordered region	Chinese	Sporadic FTD	Jiao et al. (2016)
	P34S (c.100C > T)	Hetero	Disordered region	French	FTD-ALS	Chaussenot et al. (2014)
				Italian	Sporadic ALS	Chio et al. (2015)
				Caucasian	PD	Zhang et al. (2015)
				Caucasian	AD	Zhang et al. (2015)
	A35D (c.104C > A)	Hetero	Disordered region	Italian	FTD	Zhang et al. (2015)
				Chinese	AD	Xiao et al. (2017)
	V57E (c.170T > A)	Hetero	Conserved	Chinese	FTD	Jiao et al. (2016)
			α-helix			
	G58R (c.172G > C)	Hetero	Conserved	Chinese	Autosomal dominant mitochondrial myopathy	Ajroud-Driss et al. (2015)
			α-helix			
	S59L (c.176C > T)	Hetero	Conserved	French	Late-onset FTD-ALS with cerebellar ataxia and mitochondrial myopathy	Bannwarth et al. (2014)
			α-helix			
				Spanish	FTD and FTD-ALS	Chaussenot et al. (2014)
	G66V (c.197C > A)	Hetero	Conserved	Finnish	Familial ALS	(Muller et al., 2014)
			α-helix			
				Caucasian	Late-onset SMAJ	Penttila et al. (2015)
				European	Axonal CMT2	Auranen et al. (2015)
	P80L (c.239C > T)	Hetero	Disordered region	Italian	ALS and muscle mitochondrial pathology	Ronchi et al. (2015)
				Caucasian	ALS	Zhang et al. (2015)
	Q82X (c.244C > T)	Hetero	Disordered region	Spanish	FTD	Dols-Icardo et al. (2015)
	Y92C (c.275A > G)	Hetero	Disordered region	Chinese	Sporadic ALS	Zhou et al. (2017)
	Q102H (c.306G > C)	Hetero	CHCH domain	Chinese	Sporadic ALS	Zhou et al. (2017)
	Q108X (c.322C > T)	Hetero	CHCH domain	Belgium	FTD	Perrone et al. (2017)
	Q108P (c.323A > C)	Hetero	CHCH domain	German	ALS	Lehmer et al. (2018)

Hetero, Heterozygous; *Homo*, Homozygous; PD, Parkinson’s disease; AD, Alzheimer’s disease; FTD, frontotemporal dementia; MSA, multiple system atrophy; ALS, amyotrophic lateral sclerosis; SMAJ, late-onset spinal motor neuropathy; CMT2, Charcot-Marie-Tooth disease type 2.

CHCHD10 S59L mutation was first identified in a French family with a dominant inheritance pattern (Bannwarth et al., 2014). Patients from this family had motor neuron disease, cerebellar syndrome, cognitive decline, and myopathy. Fibroblasts harvested from these patients had respiratory chain deficiency, mitochondrial ultrastructural alterations, and mitochondrial network fragmentation. Conversely, hyperfusion of mitochondria in fibroblasts derived from an ALS patient with CHCHD10 R15L has been reported (Straub et al., 2018). *CHCHD10* variants have been reported in patients with FTD, ALS, motor neuron disease, spinal motor neuronopathy, Charcot-Marie-Tooth disease type 2, and mitochondrial myopathy. Table 1 summarizes these variants.

Immunohistochemical analysis revealed that CHCHD2 and CHCHD10 are abundantly expressed in nigrostriatal dopaminergic neurons, cortical and hippocampal pyramidal neurons, and motor neurons in the anterior horn of the spinal cord, which are associated with the pathology of PD, AD, and ALS-FTD (Burstein et al., 2018; Huang et al., 2018). However, another study discovered that CHCHD10 expression is much lower in the spinal cord and sciatic nerves of mice than in the skeletal muscles (Xiao et al., 2020). In the substantia nigra of the mouse midbrain, CHCHD2 immunosignals highly overlapped with dopaminergic neurons, whereas CHCHD10 immunosignals exhibited a broad staining pattern including dopaminergic neurons (Zhou et al., 2019). Our study revealed that CHCHD2 is expressed as sufficient immunosignals in nigrostriatal dopaminergic and anterior cingulate cortex neurons (Ikeda et al., 2019). Importantly, CHCHD2 T61I mutation resulted in the loss of mitochondrial localization and large aggregation of CHCHD2, suggesting that the T61I mutation has both loss-of-function and toxic gain-of-function properties (Ikeda et al., 2019).

## 3 Physiological roles of CHCHD2 and CHCHD10

CHCHD2 and the cytochrome *c*-binding protein MICS1 stabilize cytochrome *c* in the respiratory chain complex in mouse embryonic fibroblasts and regulate electron transfer (Meng et al., 2017). CHCHD2 binding to cytochrome *c* oxidase (COX) subunit 4 was facilitated by Tyr99 phosphorylation mediated by Abl2 kinase in HEK293 cells which were stably overexpressing CHCHD2 (Aras et al., 2017), however, another study failed to detect direct CHCHD2 binding to COX subunit 4 using purified proteins (Meng et al., 2017). When HEK293 cells which stably overexpress CHCHD2 are exposed to hypoxic conditions, CHCHD2 is translocated to the nuclei where it transactivates the expression of an isoform of COX subunit 4 and itself. Although the isoform of COX subunit 4 is expressed in the lung at high levels and in the heart and brain at low levels, CHCHD2 may facilitate OXPHOS under hypoxic conditions in different tissues (Aras et al., 2013; Aras et al., 2015).

CHCHD10, which is found near the mitochondrial cristae junction, was reported to be resident in the mitochondrial contact site and cristae organizing system (MICOS) complex, containing mitofilin, MINOS1, CHCHD3, CHCHD6, APOO, and APOOL (Bannwarth et al., 2014; Koob and Reichert, 2014; Genin et al., 2018). Another study also reported that CHCHD10 was associated with optic atrophy-1 (OPA1) and the mitofilin/MICOS complexes, which helped to stabilize OPA1 and the mitofilin complexes in HEK293T cells (Liu T. et al., 2020). The stabilization of the mitofilin/MICOS complex is expected to promote the maintenance of cristae integrity and mitochondrial respiratory complex activity (Liu T. et al., 2020).

## 4 Similarities and interactions between CHCHD2 and CHCHD10

Although *CHCHD2* and *CHCHD10* mutations cause PD and ALS/FTD, respectively, these proteins have similar amino acid sequences (Imai et al., 2019b). *C. elegans* and *Drosophila* have single-copy orthologs of *CHCHD2* and *CHCHD10*, *har-1* and *CG5010*, respectively, suggesting that these two proteins diverged during the evolution into higher animals (Imai et al., 2019b, Zubovych. et al., 2010). While many studies have described the molecular relationship between CHCHD2 and CHCHD10, few have analyzed their differences in detail.

In the mitochondrion, CHCHD2 forms a homodimer with itself and a heterodimer with CHCHD10 (Meng et al., 2017; Burstein et al., 2018; Straub et al., 2018). CHCHD10 oligomers were not detected in CHCHD2 knockout HeLa and HEK293 cells, indicating that CHCHD2 is required for CHCHD10 oligomerization (Huang et al., 2018). However, in the absence of CHCHD2, CHCHD10 oligomers were detected as wild-type in mouse brains (Nguyen et al., 2022). Moreover, CHCHD2 is not required for the aggregation of CHCHD10 S59L at the mitochondria (Baek et al., 2021).

In human fibroblasts, CHCHD2 and CHCHD10 formed a 220-kDa complex, which did not contain the MICOS (Straub et al., 2018). In contrast, a super-resolution microscope analysis revealed that CHCHD2 and CHCHD10 were co-localized with the MICOS and maintained its integrity and mitochondrial cristae morphology in human neuroblastoma SK-N-SH cells (Zhou et al., 2019). These studies suggest that CHCHD2 and CHCHD10 are co-localized with the MICOS rather than bound to the MICOS components.

In HeLa knockout cells complementarily expressing CHCHD2 and FLAG-tagged CHCHD10, both CHCHD2 and CHCHD10 also interacted with and inhibited an m-AAA protease OMA1, which regulates the degradation of a mitochondrial fusion factor OPA1 (Ruan et al., 2022). However, another study found no evidence of stable CHCHD2 binding to OMA1 in HeLa cells stably expressing FLAG-tagged CHCHD2 (Liu Y. T. et al., 2020). Molecular details of CHCHD2/CHCHD10 and OMA1 are described in Section 9.

## 5 The consequence of CHCHD2 and CHCHD10 loss

CHCHD10 knockout mice had normal lifespan with no obvious mitochondrial phenotypes (Anderson et al., 2019), while conditional CHCHD10 ablation in mouse skeletal muscles impaired motor functions and caused neuromuscular junction degeneration (Xiao et al., 2020). CHCHD10 knockdown in zebrafish caused axon length reduction in motor neurons, abnormal myofibrillar structures, and motor deficits (Brockmann et al., 2018).

CHCHD2 knockout mice also showed a normal lifespan and body weight, yet they exhibited motor deficits and p62-positive *α*-synuclein aggregation in the dopaminergic neurons of the midbrain (Sato et al., 2021). Moderate degeneration of midbrain dopaminergic neurons was also observed in 120-week-old CHCHD2 knockout mice (Sato et al., 2021).

No obvious degeneration of dopaminergic neurons in the midbrain was observed in CHCHD2/CHCHD10 double knockout mice at 13 months of age (Nguyen et al., 2022); however, cardiomyopathy and a mitochondrial-integrated stress response (mtISR) (details will be described in Section 10) were observed in the heart at 9–13 weeks of age (Liu Y. T. et al., 2020).

Increased basal and maximal respiration was observed in iPS cells lacking CHCHD2 or CHCHD10, which may be a compensatory response due to compromised mitochondrial inner membrane integrity (Harjuhaahto et al., 2020). Motor neurons derived from iPS cells without CHCHD2 or CHCHD10 shared common transcriptome profiles such as reduction in *α*-amino-3-hydroxy5-methyl4-isoxazolepropionic acid-type, kainate-type, and N-methyl-d-aspartic acid-type glutamate receptor subunits, but no changes in electrophysiological properties were detected (Harjuhaahto et al., 2020).

Therefore, these reports suggest that CHCHD2 and CHCHD10 deficiency has a mild effect on mitochondria-rich tissues such as the heart, skeletal muscles, and neurons in an age-dependent manner, which appears to be milder than the consequence of pathogenic mutations described in the next section.

## 6 Pathogenicity of CHCHD10 mutations and transactive response DNA binding protein (TDP-43) accumulation

The relationship between CHCHD10 mutation and TDP-43 accumulation has been reported in various cell and animal models. CHCHD10 R15L and S59L induced the mislocalization of nuclear TDP-43 to the cytoplasm and mitochondria in NIH3T3 cells (Woo et al., 2017). In another study, CHCHD10 S59L promoted TDP-43 aggregation and mitochondrial localization in *Drosophila*, HeLa cells, and SH-SY5Y cells (Baek et al., 2021). CHCHD10 R15L or CHCHD10 S59L transgenic mice exhibited significantly increased CHCHD10 aggregation and phosphorylated TDP-43 pathology, which often co-localized within the same inclusion bodies, leading to impaired functional outcomes in long-term synaptic plasticity, motor unit physiology, and behaviors (Liu et al., 2022).

Mitochondrial myopathy and mitochondrial DNA (mtDNA) instability were observed in patients with CHCHD10 S59L mutation, but not in patients with CHCHD10 G66V mutation (Bannwarth et al., 2014; Genin et al., 2018). Fibroblasts from patients with CHCHD10 S59L revealed an impaired MICOS complex integrity while this was not observed in G66V fibroblasts (Genin et al., 2018). However, S59L and G66V fibroblasts revealed phosphorylated TDP-43 accumulation in mitochondria (Genin et al., 2018). CHCHD10 S59L knock-in mice showed neuromuscular junction and motor neuron degeneration, and TDP-43 cytoplasmic aggregates in spinal cord neurons (Genin et al., 2019). In another study, CHCHD10 S59L knock-in mice presented progressive motor deficits, myopathy, cardiomyopathy, and accelerated mortality (Anderson et al., 2019).

In contrast to the above findings, an ALS patient with CHCHD10 R15L mutation, who experienced slowly progressive limb weakness and dysphagia, meeting the El Escorial criteria for clinically probable ALS, had no TDP-43-immunopositive inclusions (Keith et al., 2020). Although many CHCHD10 inclusions were detected in this ALS patient, these inclusions did not colocalize with TDP-43 (Keith et al., 2020). CHCHD10 R15L transgenic mice, which had a shorter lifespan and degeneration of the central nervous system, skeletal muscle, and myocardium, did not have TDP-43-immunopositive inclusions, which was consistent with the pathological findings of the above patient with ALS (Keith et al., 2020; Ryan et al., 2021).

CHCHD10 protein levels are reduced in patient cells with CHCHD10 R15L or G66V (Brockmann et al., 2018). Quantitative real-time polymerase chain reaction revealed that CHCHD10 R15L expression was already suppressed at the mRNA level, yet CHCHD10 G66V expression was not (Brockmann et al., 2018). This study implies that CHCHD10 G66V causes motor neuron disease primarily through CHCHD10 haploinsufficiency, although the study did not examine the insolubility of these mutants (Brockmann et al., 2018). In contrast, CHCHD10 S59L appears to have either a loss-of-function or gain-of-toxic function phenotype (Woo et al., 2017; Anderson et al., 2019; Baek et al., 2021).

In cell and animal models of CHCHD10 S59L mutation, TDP-43 tends to accumulate in mitochondria (Woo et al., 2017; Genin et al., 2018; Baek et al., 2021), while CHCHD10 R15L mutation tends to lack TDP-43 aggregation in human and animal models (Keith et al., 2020; Ryan et al., 2021). Differences in TDP-43 pathology may be due to the properties of these mutations.

## 7 CHCHD2 mutation promotes the accumulation of CHCHD10 and *α*-synuclein

CHCHD2 is preferentially stabilized by the loss of mitochondrial membrane potential, and CHCHD2 T61I and wild-type (WT) form a heterodimer with CHCHD10 in HeLa, HEK293 cells, and human primary fibroblasts (Huang et al., 2018). There were no changes in CHCHD2 protein and mRNA levels in fibroblasts from patients with CHCHD2 T61I, yet mitochondrial cristae disorganization was observed (Mao et al., 2019). Overexpression of CHCHD2 T61I in SH-SY5Y cells, however, resulted in no mitochondrial respiratory dysfunction (Mao et al., 2019). Overexpressed FLAG-CHCHD2 T61I increased CHCHD10 interaction in HEK293T cells, yet CHCHD10 levels decreased in the T61I patient fibroblasts (Mao et al., 2019) whereas FLAG-tagged CHCHD2 T61I induced CHCHD2 WT insolubility in human fibroblasts (Cornelissen et al., 2020). Consistent with this observation, insoluble CHCHD2, CHCHD10, and *α*-synuclein were detected in the brain of a PD patient with CHCHD2 T61I mutation (Ikeda et al., 2019), and CHCHD2 T61I was also co-localized with Lewy bodies (Ikeda et al., 2019). CHCHD2 T61I transgenic mice, which developed motor defects at 1 year of age, also had insoluble accumulations of *α*-synuclein and phospho-α-synuclein in the brain compared to WT littermates (Kee et al., 2022). Moreover, radioimmunoprecipitation assay-buffer insoluble proteins were increased in the brains of CHCHD2 T61I transgenic mice, with most insoluble proteins detected being mitochondria-associated proteins (Kee et al., 2022). This study indicates that the CHCHD2 T61I mutation affects the solubility of mitochondrial proteins (Kee et al., 2022).

R145Q or Q126X mutations in isogenic human embryonic stem cells and differentiated neural progenitor cells resulted in impaired mitochondrial function, reduced CHCHD2 and MICOS complex components, and near-hollow mitochondria with reduced cristae (Zhou et al., 2019). In addition, these CHCHD2 mutations lost their interaction with CHCHD10, while transient knockdown of either CHCHD2 or CHCHD10 reduced the MICOS complex and mitochondrial cristae (Zhou et al., 2019).

## 8 Mechanism of mitochondrial import of CHCHD2 and CHCHD10

Newly synthesized CHCHD2 and CHCHD10 polypeptides are imported to the mitochondrial intermembrane space and folded *via* the formation of disulfide bonds by the Mia40-mediated pathway (Fischer et al., 2013; Lehmer et al., 2018). CHCHD2 WT and CHCHD2 T61I lacking the putative MTS in the N-terminus were normally imported into mitochondria in human skin fibroblasts (Cornelissen et al., 2020). In contrast, mitochondrial localization was significantly abolished in CHCHD2 WT and T61I when all four cysteine residues in the CHCH domain were replaced with serine, suggesting that CHCHD2 mitochondrial targeting occurs via the Mia40/Erv1 redox-coupled thiol-disulfide exchange system utilizing four cysteine residues in the CHCH domain (Cornelissen et al., 2020).

In HeLa cells, CHCHD10 Q108P and C122R, which are ALS-associated missense mutants affecting the CHCH domain, exhibited impaired mitochondrial import (Lehmer et al., 2018). Deletion of the CHCH domain of CHCHD10 in HeLa cells almost completely prevented mitochondrial import, whereas deletion of the putative MTS did not inhibit mitochondrial import (Lehmer et al., 2018). Mia40 knockdown, which introduces disulfide bonds into CHCH domain proteins, prevented CHCHD10 import into mitochondria (Lehmer et al., 2018). In contrast, overexpression of Mia40 facilitated the import of CHCHD10 Q108P into mitochondria by promoting the formation of disulfide bonds (Lehmer et al., 2018). However, another study reported that both the deletion of the putative MTS and the disruption of the CHCH domain impaired mitochondrial import in HeLa cells (Burstein et al., 2018). The importance of the N-terminal sequence in their mitochondrial transport awaits further study.

## 9 CHCHD2 and CHCHD10 regulate mitochondrial fusion and cristae structure

The mitochondrial protease YME1L regulates OPA1, a GTPase that regulates the fusion of the outer and inner mitochondria, and maintains the cristae structure (Song et al., 2007). YME1L enhanced its protease activity by increasing chaperone-like protein P32 association, leading to OPA1 degradation. In contrast, CHCHD2 competed for P32 binding to YME1L, preventing OPA1 degradation (Liu W. et al., 2020). Long forms of OPA1 are required for mitochondrial fusion, whereas short forms of OPA1 limit this fusion (MacVicar and Langer, 2016). The ratio of long to short forms of OPA1 was reduced in the brain mitochondria of CHCHD2 knockout mice (Sato et al., 2021).

TDP-43 and FTD/ALS-linked CHCHD10 mutations destabilized OPA1-mitofilin complex, impairing mitochondrial fusion and respiration in brains of human FTLD-TDP (frontotemporal lobar degeneration with TDP-43 inclusions) patients, TDP-43 transgenic mice, and HEK293T cells (Liu T. et al., 2020). A mitochondrial inner membrane-resident protease, OMA1 cleaved to the long-form of OPA1, causing mitochondrial cristae disruption in *CHCHD2/CHCHD10* double knockout mice and *CHCHD10 S59L* knock-in mice (Liu Y. T. et al., 2020). CHCHD2 and CHCHD10 interacted with OMA1 and suppressed its enzyme activity, preventing OPA1 from being processed for mitochondrial fission in HeLa cells stably expressing CHCHD2 and FLAG-tagged CHCHD10 (Ruan et al., 2022), although another study failed to detect the stable binding of CHCHD2 with OMA1 in HeLa cells stably expressing FLAG-tagged CHCHD2 (Liu Y. T. et al., 2020).

In response to expression of CHCHD10 S59L, PINK1 accumulates and activates on the mitochondria, leading to the phosphorylation of its downstream targets, including mitofusin, mitofilin, and Parkin. The phosphorylation of these proteins causes mitochondrial fragmentation, and excessive mitophagy in HeLa cells and *Drosophila* (Baek et al., 2021). In summary, CHCHD2 and CHCHD10 may affect OMA1 and PINK1 activities that regulate mitochondrial morphology and mitophagy.

## 10 Dysfunction of CHCHD2 and CHCHD10 induces mtISR

In *CHCHD10 S59L* knock-in mice, CHCHD10 and CHCHD2 accumulate in affected tissues including the midbrain, spinal cord, and heart, resulting in mtISR mediated by mechanistic target of rapamycin complex 1 (mTORC1) activation (Anderson et al., 2019). CHCHD10 S59L induced mtISR, but not CHCHD10 loss, and involved the upregulation of one-carbon and serine metabolism, and downregulation of OXPHOS (Anderson et al., 2019). Another study discovered that cardiomyopathy and mtISR were induced in *CHCHD2/CHCHD10* double knockout mice and *CHCHD10 S59L* knock-in mice, but not in single knockout mice (Liu Y. T. et al., 2020).

A multi-omics study, in which transcriptomics, metabolomics, proteomics, and molecular and biochemical analyses were performed using the *CHCHD10 S59L* knock-in mouse heart, revealed that mtISR is associated with CHCHD2 and CHCHD10 aggregation and leads to metabolic rearrangement, including the activation of serine biosynthesis and one-carbon metabolism (Sayles et al., 2022). OXPHOS deficiency was preceded by mtISR and metabolic reprogramming (Sayles et al., 2022). Although one-carbon metabolism initially promotes glutathione production in response to proteotoxic stress by CHCHD2/CHCHD10 aggregation, chronic metabolic reprogramming by mtISR led to taurine depletion, nucleotide imbalances, mtDNA depletion, and eventually cardiac failure (Sayles et al., 2022).

Another study using fibroblasts from patients with sporadic ALS with CHCHD10 R15L under galactose-treated conditions, which evokes energetic stress, discovered similar changes in one-carbon metabolism (Straub et al., 2021). In this setting, mTORC1 downregulation was observed, which is in contrast to the study by Anderson et al., suggesting that metabolic reprogramming is independent of mTORC1 pathway activation (Anderson et al., 2019).

Analysis of the heart mitochondria in *CHCHD10* knock-in mice of two different pathogenic mutations G58R and S59L, revealed that G58R forms large punctate aggregates while S59L forms filamentous aggregates. In contrast to S59L, G58R is not biochemically insoluble, but induces OMA1 activation more strongly than S59L, suggesting that these two mutants have different toxic conformations (Shammas et al., 2022). Although *CHCHD10 G58R* or *S59L* knock-in mice and *CHCHD2/CHCHD10* double knockout mice reflect the consequences of toxic gain-of-function and loss-of-function in these genes, respectively, these mice exhibit more or less mtISR (Anderson et al., 2019; Liu Y. T. et al., 2020; Sayles et al., 2022; Shammas et al., 2022). These observations raise the possibility that disease-associated variants, including poorly-characterized variants, are classified as either toxic gain-of-function or loss-of-function mutations, which may be a factor in disease development differences; although *CHCHD2* or *CHCHD10* single knockout mice exhibited much weaker phenotypes (Burstein et al., 2018; Anderson et al., 2019; Liu Y. T. et al., 2020; Sato et al., 2021; Xia et al., 2022).

## 11 CHCHD2 and CHCHD10 are associated with mitochondrial unfolded protein responses

RNA sequencing of patient fibroblasts carrying the CHCHD10 R15L mutation revealed a mitochondrial complex I deficiency, resulting in an increase in the NADH/NAD^+^ ratio and a downregulation of the tricarboxylic acid cycle (Straub et al., 2021). The energy deficiency by galactose treatment increased the adenosine monophosphate (AMP)/ATP ratio, which activated AMP-activated protein kinase, leading to the activation of catabolic pathways and the downregulation of mTORC1 pathway (Straub et al., 2021). Metabolic dysregulation also resulted in endoplasmic reticulum stress, mtUPR, and autophagy activation (Straub et al., 2021). These responses are probably part of mtISR described above.

In contrast, CHCHD2 was shown to mediate mtUPR to protect mitochondrial stress in MELAS (mitochondrial myopathy, encephalopathy, lactic acidosis, and stroke-like episodes) cybrid cells (Aras et al., 2020). Under endoplasmic reticulum stress conditions, CHCHD2 was preferentially concentrated in the nucleus, which induced mtUPR through ATF5 to mitigate mitochondrial stress in response to the above states (Aras et al., 2020). Further analysis is required to reconcile these seemingly contradictory results.

## 12 PD-associated PINK 1-Parkin signaling and CHCHD2/CHCHD10

There was no evidence that CHCHD2 deficiency exacerbated the mitochondrial phenotype caused by PINK1 or Parkin loss, both of which are autosomal recessive forms of PD causative genes and are thought to be involved in mitophagy for removing damaged mitochondria (Meng et al., 2017). However, mitochondrial phenotypes in knockout flies for *CG5010*, a single ortholog of *CHCHD2* and *CHCHD10*, were exacerbated by PINK1 or Parkin overexpression (Meng et al., 2017). Similarly, PINK1 and Parkin knockdown rescued CHCHD10 S59L-associated mitochondrial phenotypes without affecting CHCHD10 S59L aggregates and insoluble TDP-43 in *Drosophila* (Baek et al., 2021). PINK1 knockdown also rescued the fragmented mitochondrial network observed in CHCHD10 S59L patient-derived fibroblasts (Baek et al., 2021). These findings suggest that PINK1-Parkin signaling excessively removes damaged mitochondria caused by CHCHD2/CHCHD10 mutations, resulting in neurotoxicity.

## 13 Future prospects for research and therapeutic options

**FIGURE 1:**
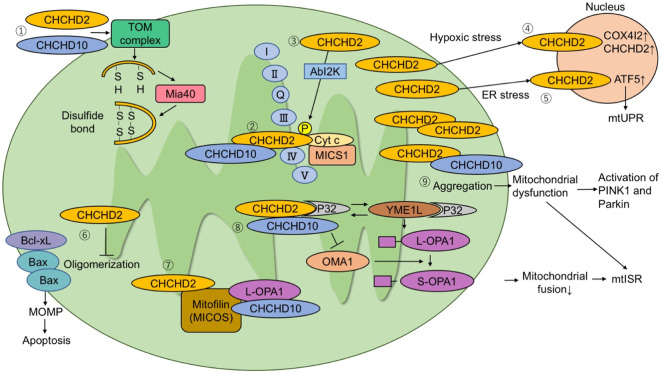
The molecular pathways proposed in literature. (1) CHCHD2 and CHCHD10 are imported to mitochondria via the TOM complex. They are folded after being imported and form a disulfide bond via Mia40. (2) CHCHD2 regulates COX activity by binding to cytochrome c, MICS1, and COX. (3) Phosphorylation of CHCHD2 by Abl2 kinase enhances affinity for COX and respiratory activity. (4) Under hypoxic stress conditions, CHCHD2 accumulates in the nucleus and functions as a transcription factor for COX4I2 and CHCHD2. (5) Under endoplasmic reticulum stress conditions, CHCHD2 activates ATF5 and induces mitochondrial unfolded protein responses. (6) CHCHD2 inhibits Bax oligomerization and subsequent mitochondrial outer membrane permeability (MOMP), suppressing apoptosis. (7) CHCHD2 and CHCHD10 bind to mitofilin to form a mitochondrial contact site and cristae organizing system (MICOS) complex. CHCHD10 binds to optic atrophy-1 (OPA1). MICOS complex is responsible for cristae morphology and maintenance in the mitochondria inner membrane. (8) P32 binds to CHCHD2 and YME1L. The interaction of P32 with YME1L accelerates the processing of L-OPA1 to S-OPA1. CHCHD2 competes with YME1L for P32 and suppresses OPA1 processing by YME1L. When L-OPA1 decreases, mitochondrial fusion is suppressed and mtISR increases. (9) When CHCHD10 aggregates in mitochondria, it causes mitochondrial dysfunction and activation of the PINK1-Parkin pathway.

**FIGURE 2:**
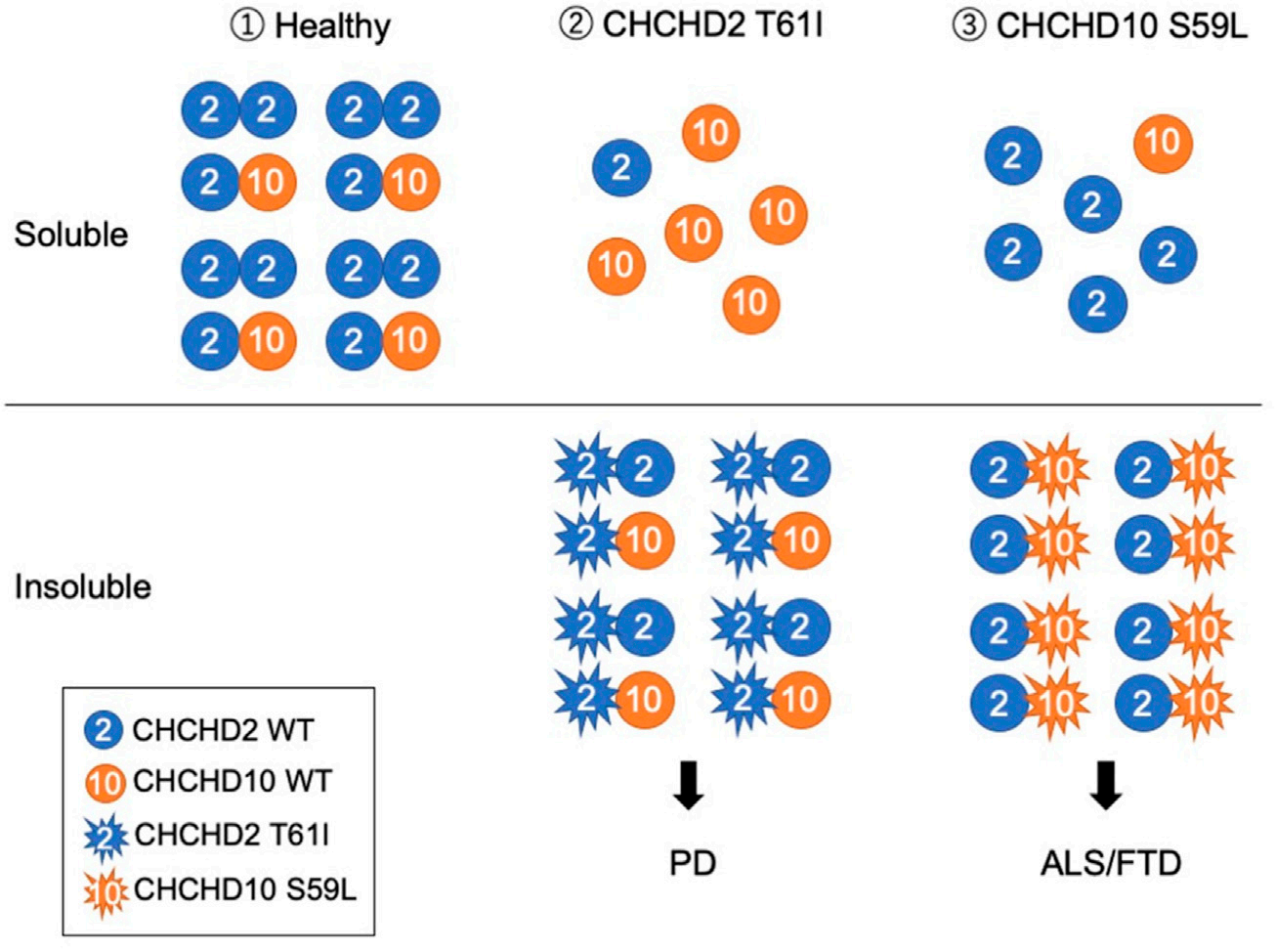
Working hypothesis that CHCHD2 T61I and CHCHD10 S59L develop different neurodegenerative diseases. (1) Under a healthy condition, CHCHD2 form a homodimer and a heterodimer with CHCHD10. (2) PD-associated CHCHD2 T61I form aggregates with CHCHD2 WT and CHCHD10 WT and insolubilize them, resulting in the induction of mtISR and the removal of functional CHCHD2 and CHCHD10. (3) ALS/FTD-associated CHCHD10 S59L form aggregates with CHCHD2 WT, resulting in insolubility together. Although the overall expression level of CHCHD10 seems to be lower than that of CHCHD2, depending on the tissue (Uhlen et al., 2015), the pathogenesis of PD and ALS/FTD may diverge, depending on which of CHCHD2 or CHCHD10 are more impaired. Mitochondrial myopathy-associated CHCHD10 G58R, which forms aggregates that do not insolubilize, affects the cristae structure and causes strong mtISR (Shammas et al., 2022). Thus, the existence of a mechanism different from the pathogenesis presented in this hypothesis is also suggested as different disease pathogenesis.

*CHCHD2* and *CHCHD10* mutations are the rare causes of PD and ALS/FTD, respectively. Similarities between CHCHD2 and CHCHD10 include their roles in OXPHOS, regulation of OMA1 and OPA1, and mtISR (Figure 1). However, it is important to resolve the functional differences between CHCHD2 and CHCHD10, when considering therapeutic strategies for PD and ALS/FTD. Hypotheses regarding the differential pathogenicity of CHCHD2 and CHCHD10 mutations to develop different neurodegenerative diseases are presented in Figure 2.

A mitochondrial protein, DELE1, cleaved by activated OMA1 is released into the cytoplasm, where the processed DELE1 interacts with and activates eIF2α kinase heme-regulated inhibitor (HRI) (Fessler et al., 2020; Guo et al., 2020). HRI phosphorylation of eIF2α induces mtISR (Fessler et al., 2020; Guo et al., 2020). CHCHD2 and CHCHD10 have been reported to interact with OMA1 to regulate OPA1 and mitochondrial morphology (Ruan et al., 2022). Appropriate regulation of OMA1 may relieve mitochondrial dysfunction, and myocardial and neurological abnormalities caused by CHCHD2 and CHCHD10 mutations (Shammas et al., 2022), but chronic mtISR by OMA1 activation may increase the risk of disease development and pathology exacerbation (Sayles et al., 2022).

An optogenetic approach in *Drosophila* lacking CG5010 demonstrated a therapeutic trial. A light-driven proton transporter, Delta-rhodopsin, was introduced into the *Drosophila* mitochondrial inner membrane (Imai et al., 2019a). Loss of CG5010 resulted in decreased ATP production, increased mitochondrial peroxide production, and decreased calcium ion buffering activity at dopaminergic terminals, which were ameliorated by proton gradient regeneration through the mitochondrial inner membrane mediated by light-stimulated Delta-rhodopsin (Imai et al., 2019a). In addition, Delta-rhodopsin suppressed *α*-synuclein aggregation, dopaminergic neuron loss, and lipid peroxidation of brain tissue caused by CHCHD2 deficiency, and improved motor behavior (Imai et al., 2019a). Because protons have radical scavenging activity, proton gradient regeneration by Delta-rhodopsin increased ATP production by restoring mitochondrial membrane potential and caused antioxidant stress effects (Imai et al., 2019a).

The fact that *CHCHD2/CHCHD10* double knockout mouse exhibits mtISR, but not single knockout mouse, suggests that CHCHD2 and CHCHD10 have complementary functions, at least in mice. In contrast, phenotypes in the central nervous system are relatively weaker than myocardial phenotypes in these mice and do not reproduce the pathophysiology of neurodegenerative diseases. One solution to overcome these difficulties may be the use of human samples and disease-associated iPS cell technology. New models should be developed to elucidate the functional differences between CHCHD2 and CHCHD10 and the pathomechanisms of diseases associated with CHCHD2 and CHCHD10.

## Abbreviations

ATP, adenosine triphosphate; AMP, adenosine monophosphate; ALS, amyotrophic lateral sclerosis; AD, Alzheimer’s disease; COX, cytochrome c oxidase; CHCHD, coiled-coil-helix-coiled-coil-helix domain; FTD, frontotemporal lobe dementia; HRI, heme-regulated inhibitor; iPS, induced pluripotent stem; MTS, mitochondrial targeting sequence; mtISR, mitochondrial-integrated stress response; MICOS, mitochondrial contact site and cristae organizing system; OXPHOS, abrOxidative phosphorylation; OPA1, optic atrophy-1; OMA1, overlapping activity with m-AAA protease; PD, Parkinson’s disease; TDP-43, transactive response DNA binding protein; WT, human wild-type; mtDNA, mitochondrial deoxyribonucleic acid.
